# Selection of SARS-CoV-2 main protease inhibitor using structure-based virtual screening

**DOI:** 10.4155/fmc-2020-0380

**Published:** 2021-11-24

**Authors:** Abdulrahim R Hakami, Ahmed H Bakheit, Abdulrahman A Almehizia, Mohammed Y Ghazwani

**Affiliations:** ^1^Department of Clinical Laboratory Sciences, College of Applied Medical Sciences, King Khalid University, Abha, 61481, Saudi Arabia; ^2^Department of Chemistry, Faculty of Science & Technology, Al-Neelain University, Khartoum, 11121, Sudan; ^3^Department of Pharmaceutical Chemistry, College of Pharmacy, King Saud University, Riyadh, 11451, Saudi Arabia; ^4^Department of Pharmaceutical Chemistry, Drug Exploration & Development Chair, College of Pharmacy, King Saud University, Riyadh, 11451, Saudi Arabia; ^5^Department of Pharmaceutics, College of Pharmacy, King Khalid University, Abha, 61421, Saudi Arabia

**Keywords:** inhibitors, nonstructural proteins, protease, SARS-CoV-2

## Abstract

**Background:** Conserved domains within SARS coronavirus 2 nonstructural proteins represent key targets for the design of novel inhibitors. **Methods:** The authors aimed to identify potential SARS coronavirus 2 NSP5 inhibitors using the ZINC database along with structure-based virtual screening and molecular dynamics simulation. **Results:** Of 13,840 compounds, 353 with robust docking scores were initially chosen, of which ten hit compounds were selected as candidates for detailed analyses. Three compounds were selected as coronavirus NSP5 inhibitors after passing absorption, distribution, metabolism, excretion and toxicity study; root and mean square deviation; and radius of gyration calculations. **Conclusion:** ZINC000049899562, ZINC000169336666 and ZINC000095542577 are potential NSP5 protease inhibitors that warrant further experimental studies.

SARS coronavirus 2 (SARS-CoV-2) causes a highly infectious disease called COVID-19, which emerged in December 2019 [1]. This virus started a global pandemic that has had an exceptionally negative impact on economies and healthcare systems. Lessons learned from SARS and Middle East respiratory syndrome were not enough, and weaknesses in healthcare facilities and preparedness for such crises must be improved significantly [2].

Conserved regions within viral nonstructural proteins represent key targets for the design of wide-spectrum inhibitors [3]. An essential protease for the life cycle of coronaviruses is the NSP5 protease [4]. The NSP5 protease of human coronavirus HKU1 and human coronavirus OC43 has been shown to aid replication of murine hepatitis virus A59, a group 2 coronavirus [5], which suggests that the main protease (M^pro^) is conserved, with similar functionality among coronavirus species. In avian gamma coronavirus, NSP5 has RNA-binding properties [6]. These findings provide novel information about the M^pro^ of coronaviruses. The NSP5 protease exists in both monomer and homodimer states, with the latter possessing catalytic activity [7]. In a post-translational modification, the NSP5 M^pro^ 3C-like protease (3CLpro) cleaves 11 sites in virus-encoded polyprotein 1ab and 1a [8].

The NSP5 protease contains two chymotrypsin-like domains and a unique third domain that might play a role in the dimerization of the enzyme [9,10]. The dimerization interface of NSP5 protease and its enzyme activity are the main targets of inhibitors. Targeting serine residues (positions 139, 144 and 147) that are adjacent to the enzyme's active site provides an effective approach for inhibiting the dimerization and catalytic activity of NSP5, especially serine at position 149 [11]. The crystal structure of human coronavirus NL63 with the Michael acceptor inhibitor N3 offers useful information for designing a broad-spectrum NSP5 inhibitor [12]. In human coronavirus OC43 and murine hepatitis virus, Y134 is a key amino acid determinant, and novel temperature-sensitive mutations (S133A and F219L) have previously been documented [5,10]. The catalytic dyad of NSP5 in coronaviruses involves the residues His41 and Cys145.

The current treatment protocols include drugs that were designed to treat viral diseases other than COVID-19 [13–15]. Antiviral agents specific to COVID-19 are indeed a global requirement. The lack of specific antiviral drugs for combating SARS-CoV-2 has led researchers to conduct randomized clinical trials on COVID-19 patients using previously approved drugs, such as HIV protease inhibitors [16]. In this study, the authors aimed to screen the ZINC database for potential NSP5 protease inhibitors using structure-based virtual screening and then evaluate them using molecular dynamics (MD) simulations.

## Methods

### Database used

The ZINC database is used to screen for chemical compounds with inhibitory potential against enzymes and other targets in a specific fashion [17,18]. A comprehensive library of antiviral compounds containing 13,840 potential antiviral phytochemicals and synthetic compounds was generated from the ZINC database using multiple ligand file formats (e.g., .sdf). Adjustment of hydrogens and lone pairs was allowed for all molecules, and charges were performed using Merck molecular force field 94x when required. After protonation of the molecules and by using a root mean square (RMS) gradient of 0.1 kcal/mol/Å, geometry minimization was performed on the molecules to optimize the arrangements [19]. Following molecule optimization, the file was saved in .mdb format (molecular operating environment [MOE] database) for subsequent virtual analysis.

### Structure-based virtual screening

The complex structure of SARS-CoV-2 NSP5 protease with boceprevir provides a basis for the detection of lead inhibitors via screening in silico. To perform virtual screening, an in-house database of potential binding compounds was utilized for molecular docking simulations via the MOE software [20] suite 910. The SARS-CoV-2 3CLpro model (Protein Data Bank [PDB] ID: 7brp, chains A and B) was used for molecular docking. PDB code 7brp is a polyprotein replicase with a resolution of 1.80 Å that cleaves the C-terminus at 11 sites. The boceprevir ligand of 7brp was used as a control. To increase the selectivity of the compounds, MOE was used to dock the selected hits at the M^pro^ binding site. Before the preliminary docking protocol was applied, the selected ligand was removed from the complex protein crystal structure, and the docking protocol was then repeated on the protein binding site, thus validating the procedure. The RMS deviation (RMSD) between cocrypted and redocked ligands was measured using MOE scientific vector language script at 1.86 Å [21]. The docking was successful in reproducing a determined binding mode for the intended protein–ligand complex.

The target protein's crystal structure was protonated using the Protonate 3D method [22], and energy minimization was accompanied by the MOE-implemented AMBER12 force field. To specifically identify the active site, the grid was set to match hits in the PDB. The triangular matcher algorithm was used as a placement method during docking. The London dG scoring and generalized Born volume integral/weighted surface area dG rescoring functions were also used. In a given binding site, all compounds were docked. Next, a protein–ligand interaction fingerprint (PLIF) module in MOE was applied to fingerprint the binding interaction of crucial residues between compounds and protein. More associations and binding poses were inspected visually using MOE 2015.10 (Chemical Computing Group, Montreal, Canada). Based on the binding profiles, five compounds from the authors' database were subjected to MD simulation.

### PLIF analysis

PLIF analysis was performed using the PLIF module in MOE 2015.10. This analysis provides valuable information regarding protein–ligand interactions by converting 3D structural binding information into a 1D binary string [23]. Protein preparation steps were first performed on the five selected proteins. These steps included the addition of hydrogen atoms, bond order assignments, disulfide fixation and protonation state assignment followed by energy minimization, which was performed utilizing the AMBER10:extended Hückel theory force field, followed by superposing the protein structures. Finally, the interaction fingerprints were generated by labeling all common binding site residues in the five proteases. These fingerprints provide valuable information that can be used for pharmacophore model generation, virtual screening and clustering.

### MD simulations

MD simulation was performed using the NAMD engine [24], which is integrated into the MOE software. The AMBER12:extended Hückel theory all-atom optimized potentials for liquid simulations force field (group II ion and group VIII parameters) [25] was applied, as it is often used to describe macromolecular systems. The system was solvated and neutralized by adding Cl^-^ or Na^+^ ions and water molecules, and the total number of solvent molecules was 9295 (equivalent to 1.023 g/cm^3^). A periodic boundary condition was utilized, and the box size was 38.29 × 38.44 × 26.57 Å. Following steepest descent geometry optimization, an equilibration of 100 steps was done in the constant pressure/constant temperature/constant number of particles ensemble with particle mesh Ewald for efficient full electrostatics. The SHAKE algorithm was utilized to ensure equilibrium values for all bonds involving hydrogen atoms [26]. Finally, the whole system was subjected to MD production runs at a temperature of 300 K for 100 ns in the absolute temperature/number of particles/volume ensemble.

For the last 100-ns MD production round, an integration time step of 2.0 fs was carried out, and the trajectories were captured after every 1 ps. The simulation analysis and visualization molecular dynamic tool use graphic processing units to accelerate the calculations. To predict the grounds for compound stability, RMSD, RMS fluctuation (RMSF), radius of gyration (RoG) and hydrogen bond stabilization were determined in the simulated systems. Prism 8 (GraphPad Software, CA, USA) was used to plot the graphs.

### Free energy calculations

Calculations of the binding free energy for the ligand-protein complex were estimated from the MD production trajectories. The molecular mechanics-generalized Born surface area/molecular mechanics-Poisson–Boltzmann surface area approach incorporated into AmberTools20 was utilized for the free energy calculations [27]. The following method was carried out to estimate the binding free energy (ΔG_bind_) within each ns [28]:

(Eq. 1) $$\Delta {\text{G}}_{\text{bind}}={\text{G}}_{\text{complex}}-{\text{G}}_{\text{receptor}}-{\text{G}}_{\text{ligand}}$$

The absolute free energy (G) for every case of the complex, receptor and ligand was calculated utilizing the formulas:

(Eq. 2) $$\text{G}={\text{E}}_{\text{MM}}+{\text{G}}_{\text{sol}}-\text{TS}$$

(Eq. 3) $${\text{G}}_{\text{sol}}={\text{G}}_{\text{sol}-\text{ele}}+{\text{G}}_{\text{SASA}}$$

in which E_MM_ is the molecular mechanics energy (sum of internal, electrostatic and van der Waals energy terms), G_sol_ is the desolvation free energy, G_sol-ele_ is the electrostatic solvation component and G_SASA_ is the non-electrostatic solvation component. Runs of 100 ns were utilized for normal mode calculations for amino acid residues around the ligand of less than 12 Å.

### Absorption, distribution, metabolism, excretion and toxicity studies

Absorption, distribution, metabolism, excretion and toxicity (ADMET) characteristics were foreseen using the ADMET descriptors in BIOVIA Discovery Studio 4.5 (Dassault Systèmes, Accelrys, CA, USA). This module uses six mathematical models to properly quantify and predict ADMET properties [29]. The ADMET absorption model can predict human intestinal absorption (HIA) after oral administration. The 95% and 99% confidence ellipses in the ADMET 2D polar surface area (2D PSA) and ADMET AlogP98 plane define the absorption levels of the HIA model [30]. The ellipses highlight the domains where compounds with excellent absorption are likely to be selected. The upper limits of the 2D PSA values for the 95% and 99% confidence ellipsoids are 131.62 and 148.12, respectively. The ADMET aqueous solubility model predicts the solubility of each compound in water at 25°C. This model is based on a genetic partial least squares method using 784 compounds in which solubility was measured experimentally [31].

The ADMET blood–brain barrier (BBB) model predicts the BBB penetration of a ligand following oral administration. This model originated from a quantitative linear regression study to predict BBB penetration and the 95% and 99% confidence ellipses analogous to those of HIA in the plane of ADMET 2D PSA and ADMET AlogP98. These values were obtained from many molecules that enter the CNS following oral administration [32].

An ADMET plasma protein binding model was used to predict whether a compound has high binding characteristics to blood carrier proteins. Calculations are based on AlogP98 and 1D symmetry to two marker compounds. One group is used to mark the binding at a level of ≥90%, whereas the other group flags the binding at a higher level of ≥95%. The predicted binding affinity is modulated based on the situations used to calculate logP [33]. The ADMET CYP2D6 binding model predicts the inhibition of the cytochrome P450 2D6 enzyme using probability speculation and the 2D chemical structure. These predictions are based on a training set of 100 molecules that have CYP2D6 inhibition characteristics. The ADMET hepatotoxicity model predicts the potential human liver toxicity of many miscellaneous compounds. The predictions are based on a model-based recursive partitioning of 382 training compounds that are toxic to the liver. Liver toxicity occurs as a result of a reaction to certain substances or drugs and can be characterized by neoplasia or elevated aminotransferase levels in a dose-dependent manner in >10% of the human population [34].

## Results & discussion

### Validation of the docking procedure

**Figure 1.**
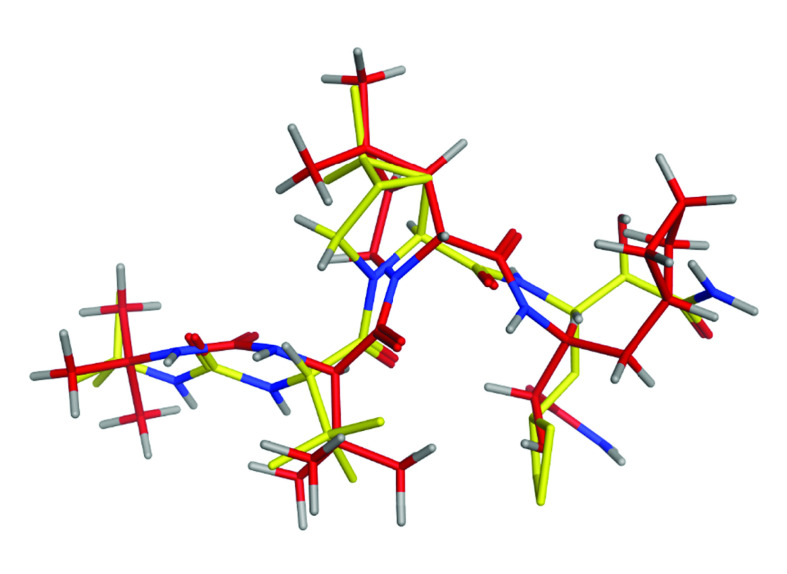
Yellow native co-crystallized ligand and red docked ligand.

The first step in the docking study was assessing the MOE-Dock program accuracy. This was performed by extracting the co-crystallized ligand from the active site and redocking within the inhibitor's binding cavity of the NSP5 protease. Initially, the RMSD value was found to be 1.86 Å (Figure 1), proving that the authors' docking procedure was valid for the studied inhibitors [35] and the MOE-Dock method was thus valid for docking these inhibitors.

### Coronavirus M^pro^ & SARS-CoV-2 M^pro^ inhibition

**Figure 2.**
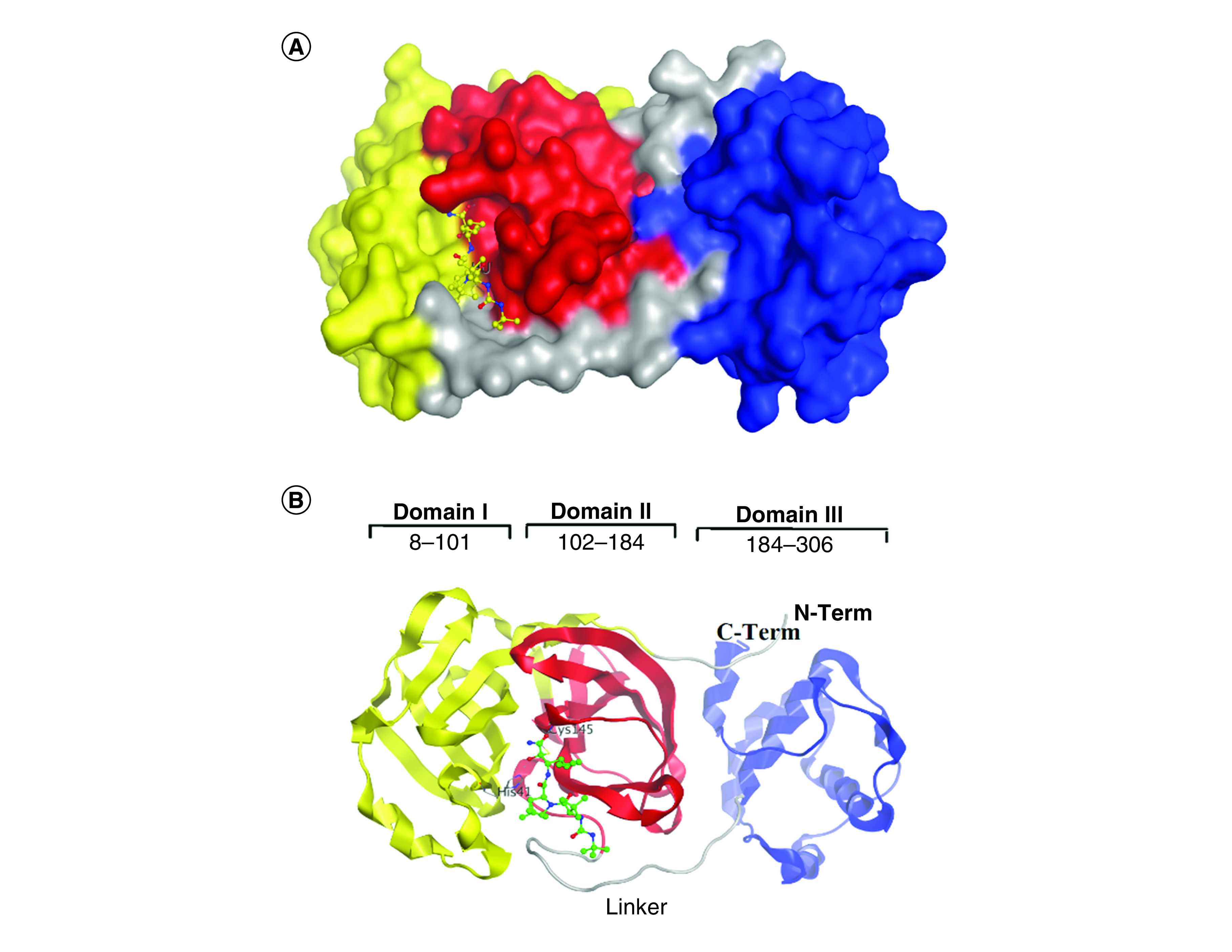
Crystal structure of SARS coronavirus 2 main protease–boceprevir. **(A)** Three domains of main protease with boceprevir. **(B)** Structural characteristics of SARS-CoV-2 M^pro^ monomer. The active site, which consists of the Cys-His dyad, lies at the interface of domains I and II. Dimerization of the protein is controlled by the linker that joins domains II and III.

According to a previous study [20], the mechanism of inhibition of boceprevir was determined in the crystal structure of SARS-CoV-2 M^pro^–boceprevir through studies of this complex at a resolution of 2.1 Å. Only one polypeptide was found in the asymmetric array. Nevertheless, two polypeptides, protomers A and B, were shown to be connected by a twofold crystallographic axis of symmetry to form a dimer (Figure 2A). On the electron density maps, residues 1–306 were visible. There were three domains within the promoter (Figure 2B). Residues 8–101 formed the first domain (domain I), whereas residues 102–184 formed domain II. Both domains were shown to have an antiparallel β-structure. Residues 201–303 formed domain III and were composed of five α-helices arranged in antiparallel spherical symmetry as well as a spacer region of short length (residues 185–200) that connected domain III to domain II. In addition, 3CLpro has a Cys-His dyad, which was found in the groove between domains I and II. In the proteolysis process, cysteine thiol serves as a nucleophile [20]. Proteolysis is initiated upon NSP5 binding with its peptide-like substrates in the S1 pocket to form a replication–transcription complex [36,37].

**Figure 3.**
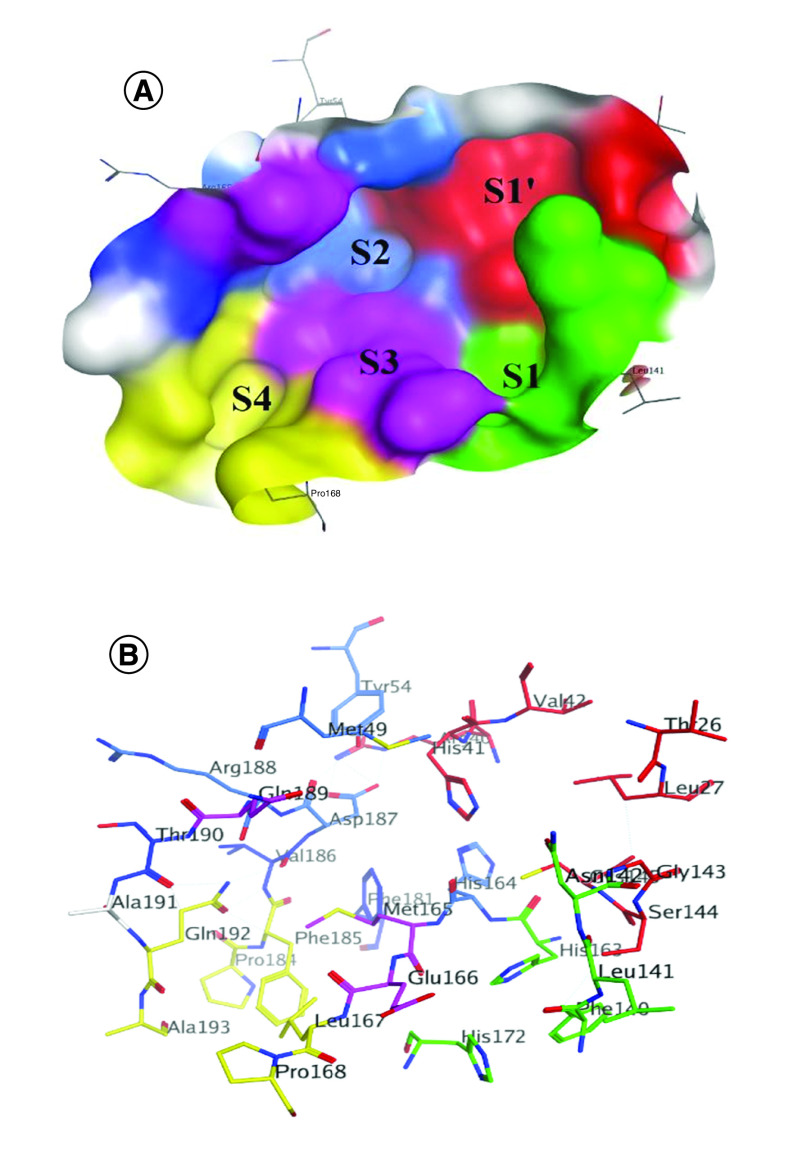
Main protease active site. Surface representation shows the binding pocket of NSP5 protease. **(A)** Substrate-binding subsites of SARS-CoV-2 NSP5 (S1′, S1, S2, S3 and S4) (Protein Data Bank ID: 7brp). **(B)** The key amino acids of the binding site are indicated in red (S1′), green (S1), blue (S2), purple (S3) and yellow (S4).

Coronavirus utilizes chymotrypsin-like activity as well as papain protease to treat and cleave viral long polyprotein precursor to afford independent functional nonstructural proteins [38]. Proteases of coronavirus species have several conserved domains. The locations of the intended subsite amino acids in the enzyme active site are known as S1, S1′, S2, S3 and S4 (Figure 3A) [39,40]. In the NSP5 active site region, Cys145, Gly143 and Ser144 contribute the S1′ residues, which also serve as oxyanion holes. The S1 group contains His163 residue, whereas Glu166 and Gln189 are located at the S2 position. The S3 subsite is completely exposed to the solvent [36,41]. Finally, the S4 site was made up of amino acids with bulky side-chains i.e. Gln189 and Pro168 (Figure 3B) [42].

### PLIF analysis

**Figure 4.**
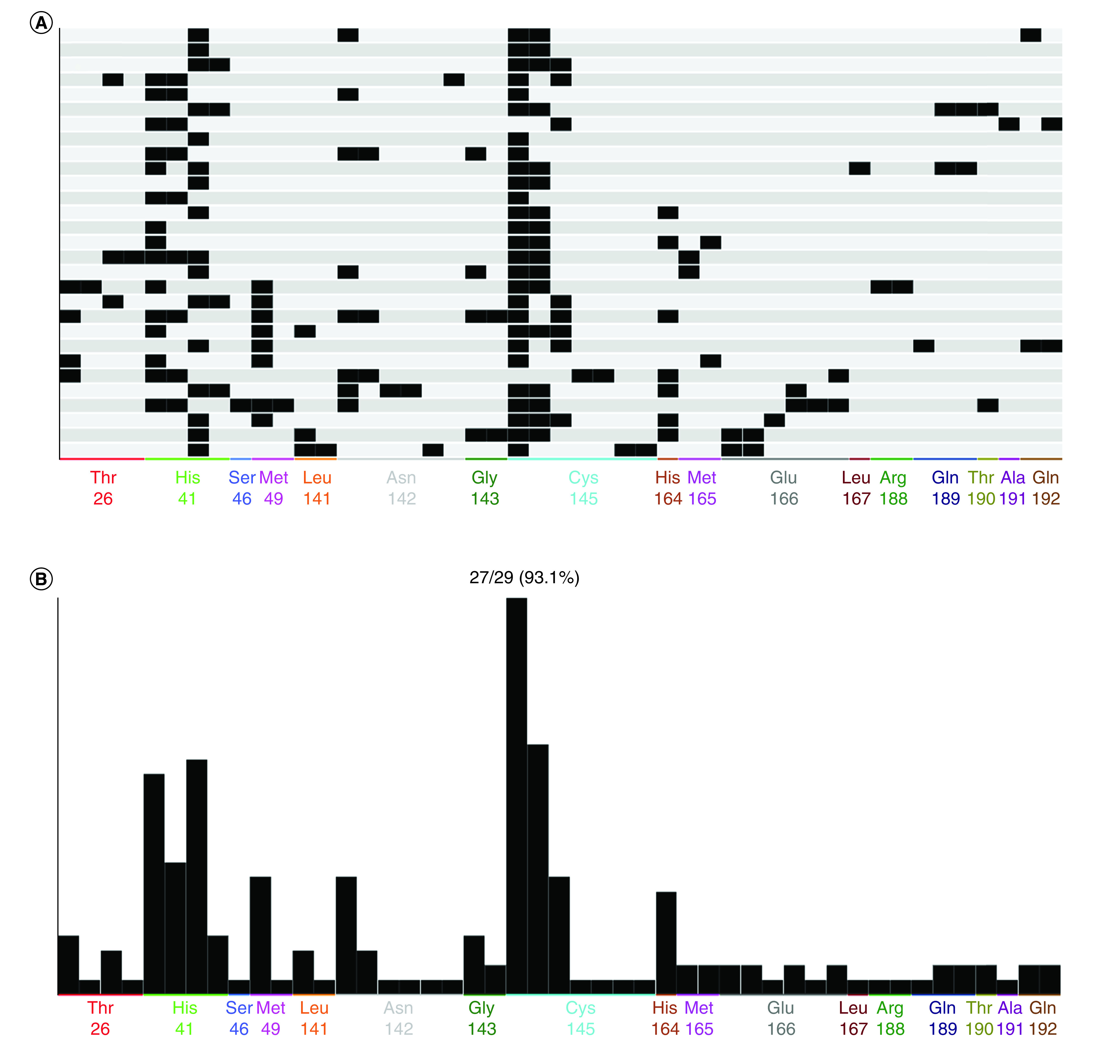
Overall protein–ligand interaction fingerprint analysis of 3C-like protease. **(A)** Matrix display of protein–ligand interaction fingerprint analysis results as a barcode. **(B)** Frequency of occurrence of fingerprint bits.

As seen in Figure 4, an automatic visualization of the crucial interactions with the residues was constructed through the utilization of PLIF analysis. The fingerprint bits showed several interactions, including ionic and surface interactions and side chain acceptor and backbone acceptor interactions. Nevertheless, the fingerprint bits revealed some important residues, such as Thr26, His41, Ser46, Met49, Leu141, Asn142, Gly143, Cys145, His164, Met165, Glu166, Leu167, Arg188, Gln189, Thr190, Ala191 and Gln192, that contributed to the protein–ligand interaction.

Within all complexes, His41 and Cyc145 demonstrated surface and side chain interactions, respectively. In addition, Cys145 was shown to display side chain acceptor, bond donor and backbone acceptor interactions in most of the complexes. Finally, the aromatic ring in His41 caused surface interactions with the ligands.

### Structure-based virtual screening

The antiviral compound library was utilized for structure-based virtual screening for SARS-CoV-2 M^pro^. The process relies on the determination of computationally suitable molecules in the 3D structure and active site of the target protein, after which these compounds are ranked according to their interaction profiles. The interaction profiles were studied using molecular docking that was carried out on NSP5 (PDB ID: 7brp) using MOE 2015.10.

The binding site of NSP5 contains a conserved Cys145 and His41 catalytic dyad with other hot spot amino acids, such as residues Met165, Glu166, Gln189 and Thr190 [19]. The docking, which is used to filter the prepared database, relies on predicting possible chemical probes with highest binding affinity to those active site residues. To cover all of the active site residues, the ligand (boceprevir) was used as a template. Of the 13,840 compounds docked, the authors selected 353 compounds with substantial affinities that achieved docking scores of less than -12.0.

**Figure 5.**
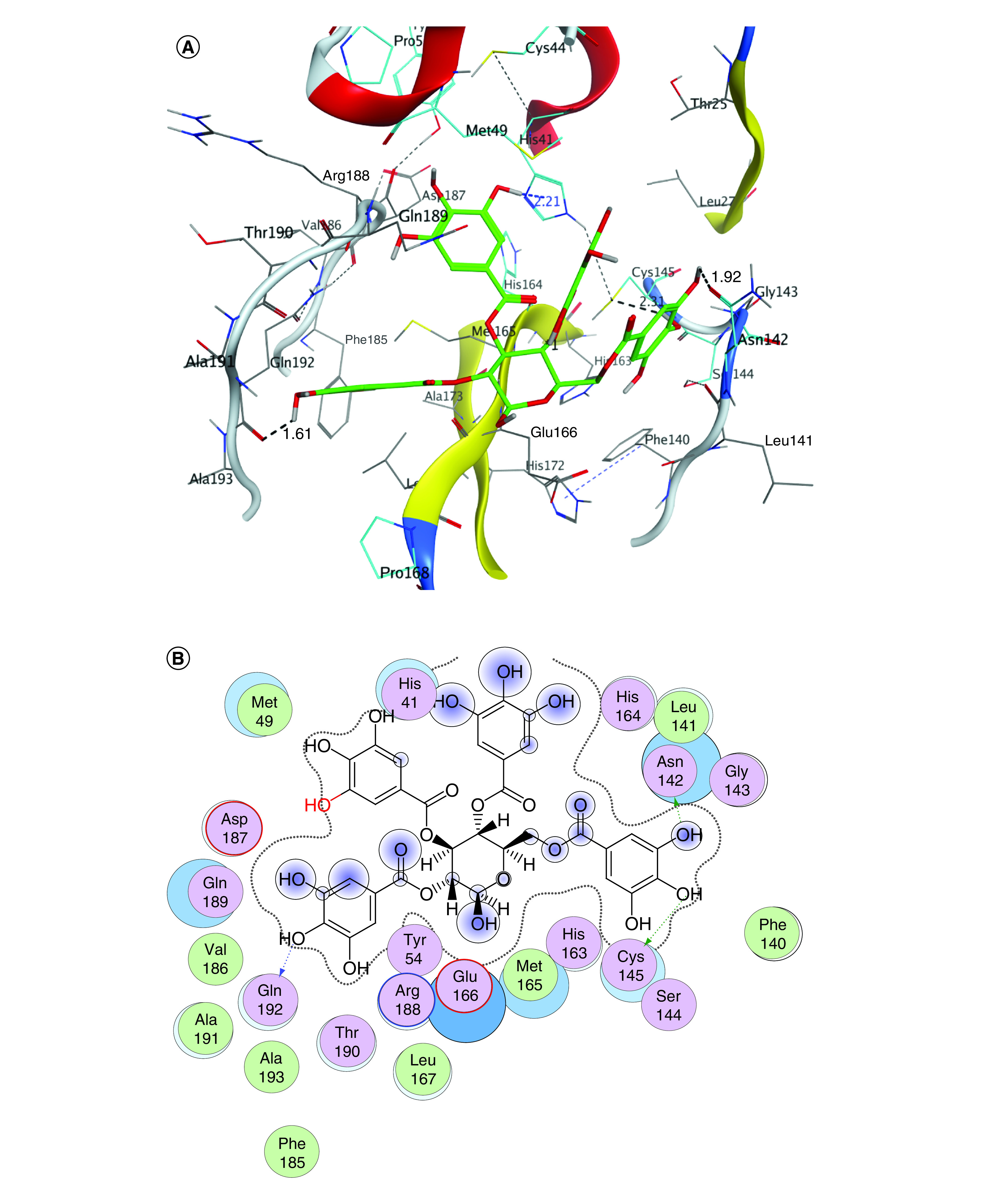
ZINC000095542577 lead compound interaction with SARS coronavirus 2 main protease enzyme. **(A)** 3D view of M^pro^ interaction with the lead compound. Green residues represent the lead compound involved in hydrogen bonding. **(B)** 2D view of M^pro^ interaction with the lead compound. M^pro^: Main protease; SARS-CoV-2: SARS coronavirus 2.

The PLIF module in MOE was used to analyze the interactions between the active site residues and selected candidate compounds. The results of candidate compound selection revealed hydrogen bond interactions with the catalytic dyad. Therefore, among the 353 compounds, ten hits were selected based on the significance of the interactions with the catalytic dyad and other active site residues. The docking scores indicated that compound **ZINC000095542577** showed the highest binding affinity compared with the others (-16.499 kcal/mol) (Figure 5). The authors observed significant interactions of all ten selected compounds via the Cys145 and His41 catalytic dyad with residues of the S1–S4 subsite pocket (Table 1). To determine the stability of the selected compounds, MD simulation of the selected hits was carried out.

**Table 1.** ZINC codes, chemical structures and interaction scores of the ten hits.

No.	ZINC code	Structure	Interaction score (kcal/mol)
1	ZINC000003583397	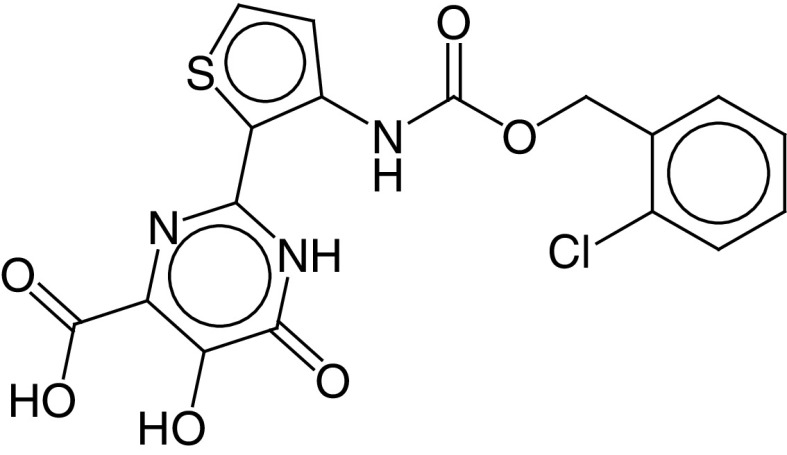	-12.06
2	ZINC000029040250	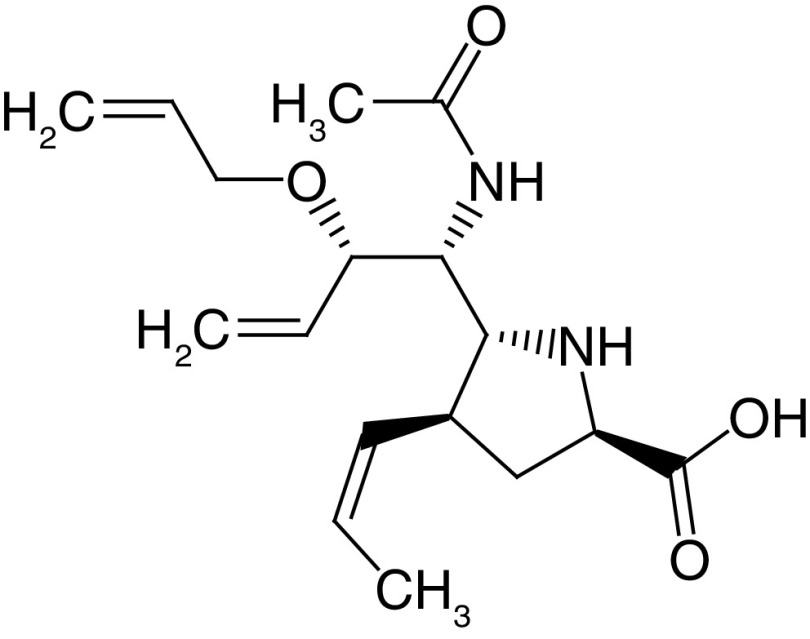	-12.11
3	ZINC000095536495	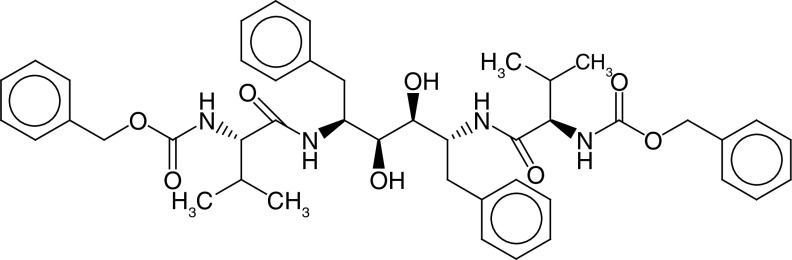	-12.61
4	ZINC000028387333	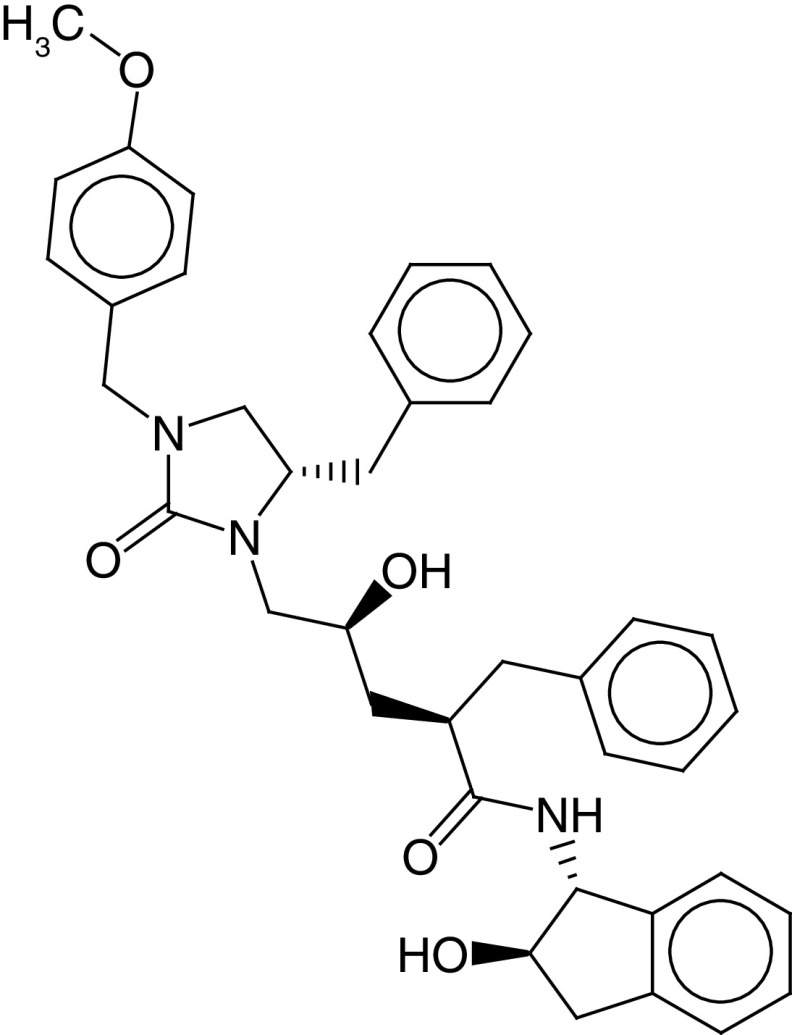	-12.84
5	ZINC000049899562	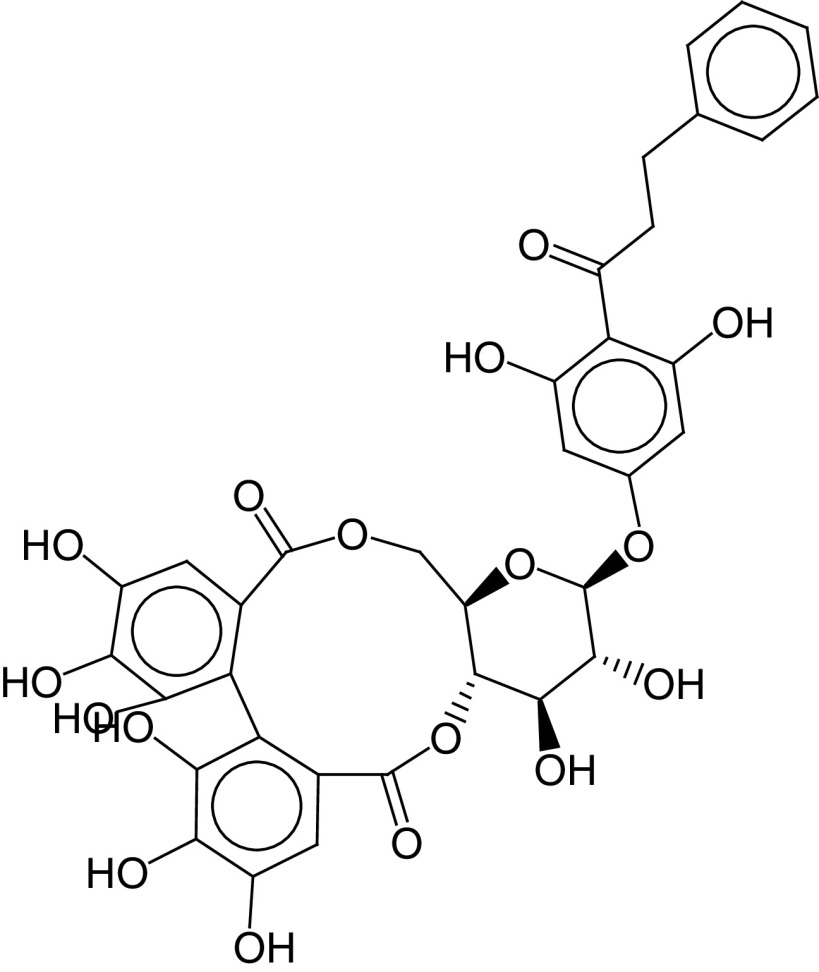	-13.42
6	ZINC000014907365	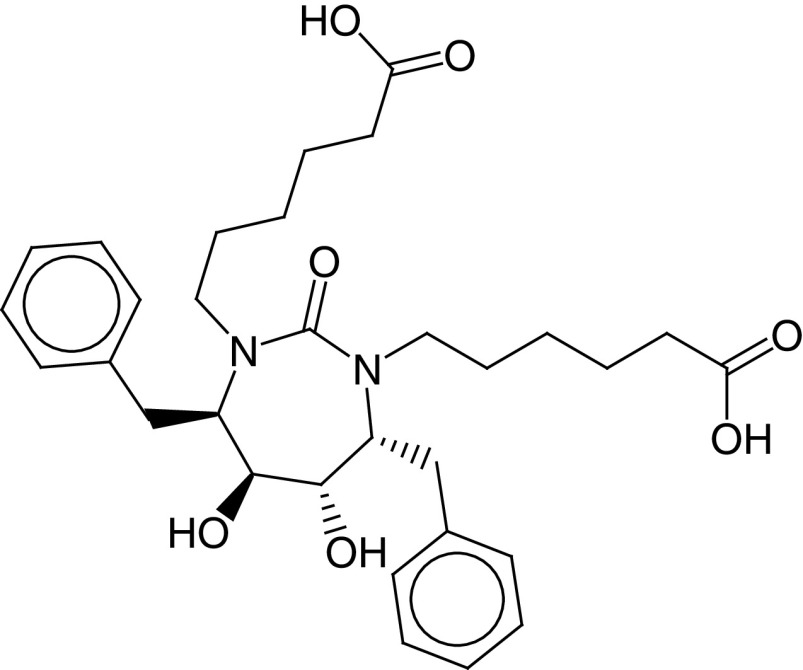	-14.58
7	ZINC000014945752	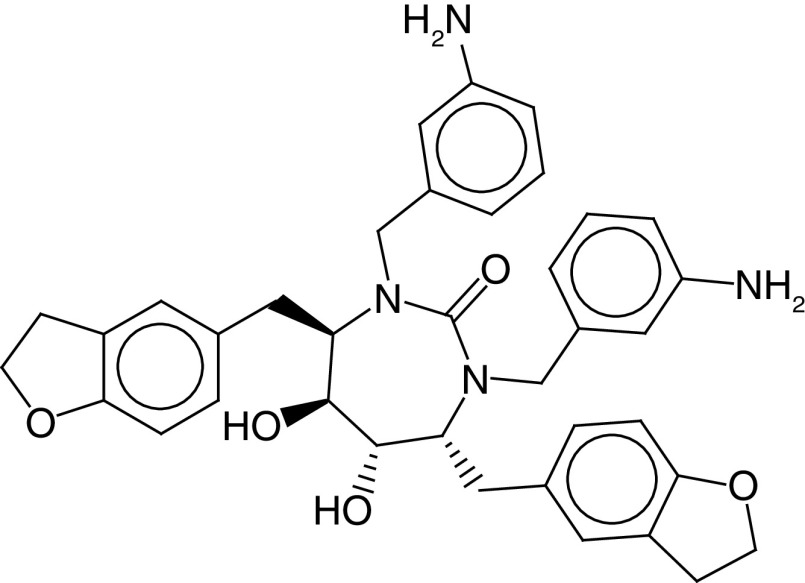	-14.89
8	ZINC000095539404	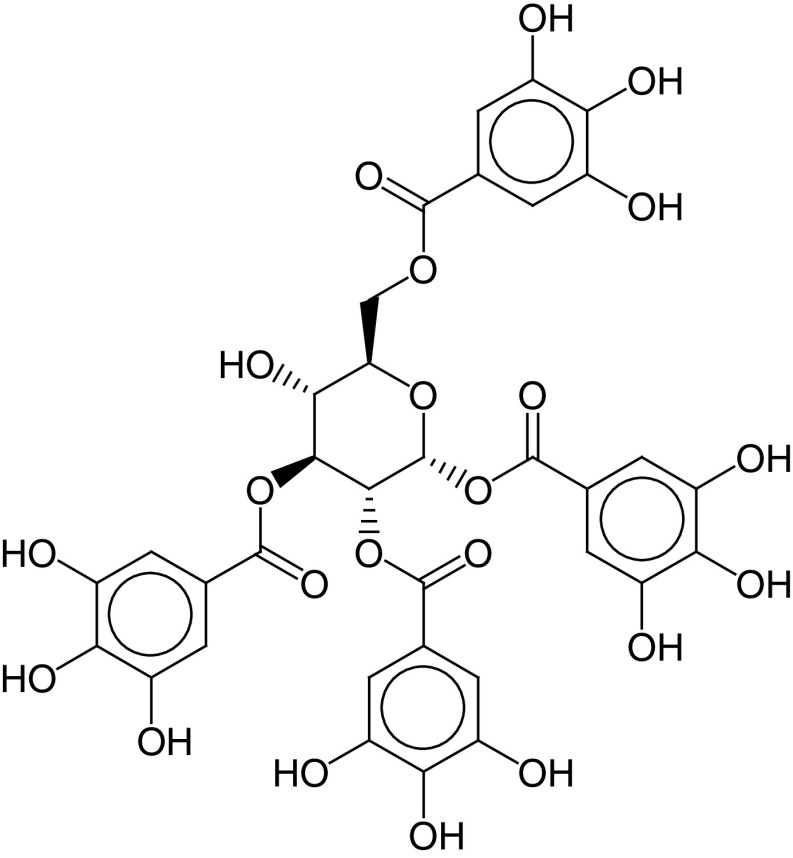	-14.91
9	ZINC000169336666	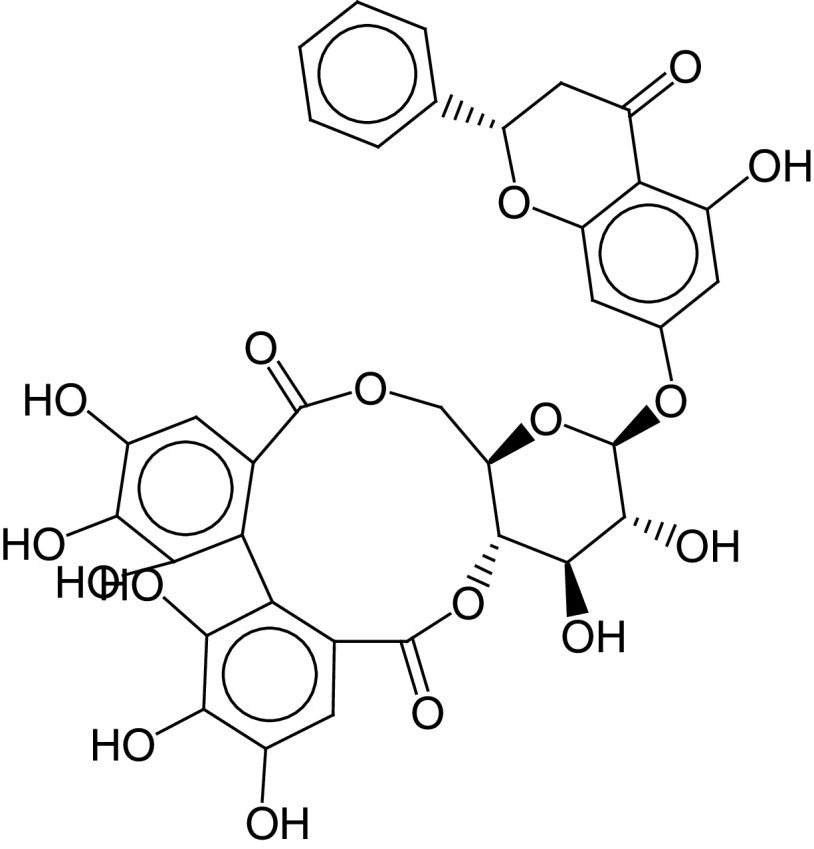	-15.13
10	ZINC000095542577	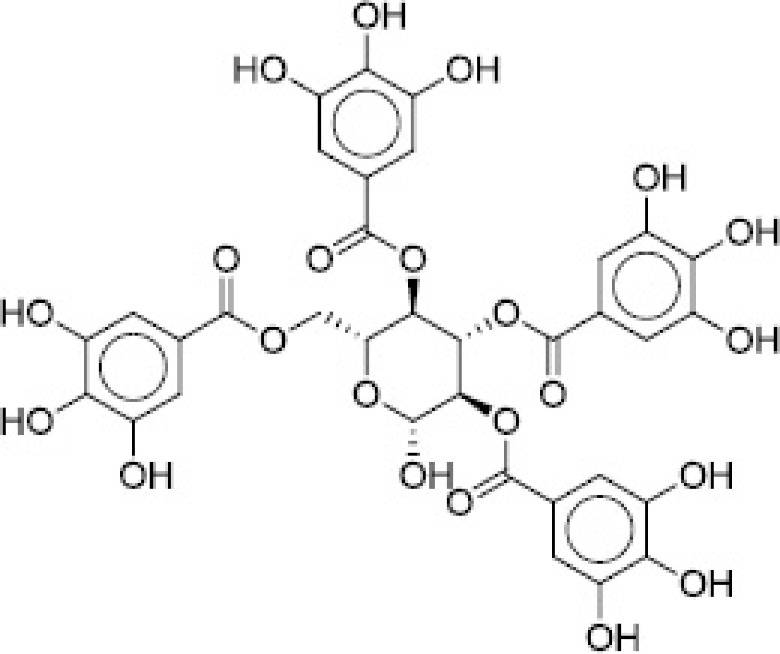	-16.50

### MD simulation

MD simulation is a solid method for studying conformational stability and protein dynamics. Five systems were selected for 100-ns simulation studies: **ZINC000169336666**, **ZINC000049899562**, **ZINC000095539404**, **ZINC000028387333** and **ZINC000095542577**.

### Binding free energy

By utilizing the MMPBSA.py method, binding free energy calculations were performed using the default settings. Table 2 shows the total binding free energy values for the final 20 ns of simulations as well as the energy contribution of each component. The results showed that **ZINC000028387333** had a higher binding affinity for M^pro^. Nevertheless, the higher binding affinity for compound **ZINC000028387333** resembled the binding affinity for the reference compound. These results are consistent with the molecular docking studies. In fact, all compounds displayed higher affinity for M^pro^, but the affinity was less than that observed for **ZINC000028387333**. Electrostatic interactions predominated the total binding energy of compound **ZINC000028387333** to the target than van der Waals interactions. However, van der Waals interactions predominated the binding energy for all the remaining complexes as well as boceprevir. The nonpolar solvation energy was similar in all complexes and favorable for binding, but the polar solvation energy was similar in **ZINC000049899562**, **ZINC000169336666** and **ZINC000095539404** complexes and dissimilar in **ZINC000095542577**, **ZINC000028387333** and **boceprevirmds** complexes.

**Table 2.** Predicted binding free energy and individual energy components for the studied molecules.

Compound	MM energy components (kcal/mol)		GBSA energy components (kcal/mol)		
	ΔE_vdw_	ΔE_ele_	ΔG_SASA_	ΔG _sol_	ΔG _binding-GBSA_
**ZINC000095542577**	-52.613	-66.358	-8.125	85.663	-110.413
**ZINC000049899562**	-48.30	-37.698	-7.124	57.688	-112.152
**ZINC000169336666**	-40.339	-34.994	-5.880	55.310	-102.553
**ZINC000095539404**	-42.475	-36.216	-6.430	57.941	-103.070
**ZINC000028387333**	-48.876	-65.359	-7.826	77.611	-123.322
Boceprevir	-52.262	-26.080	-7.475	-3.126	-125.256

GBSA: Generalized Born surface area; MM: Molecular mechanics.

### RMSD analysis

**Figure 6.**
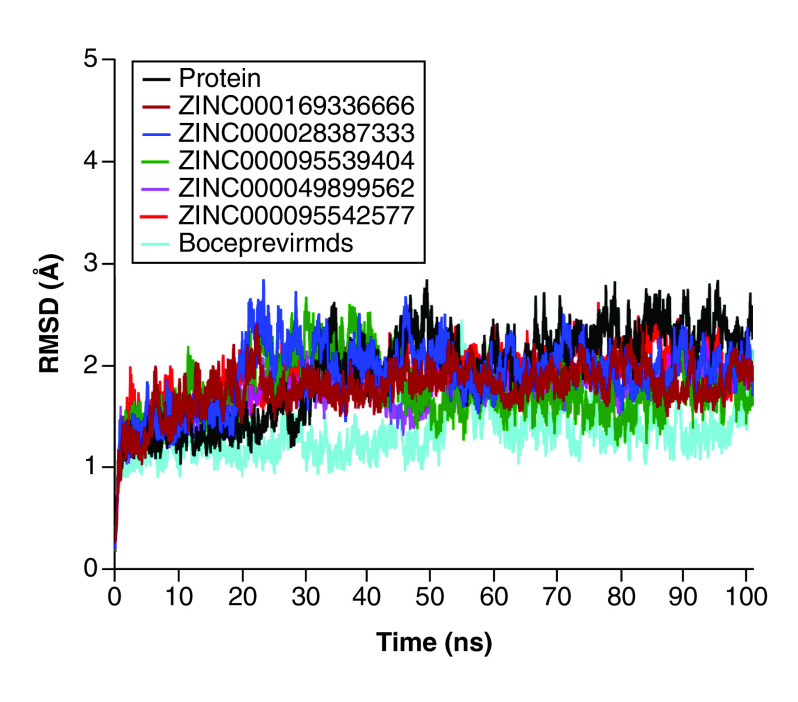
Measurements of root mean square deviation of the simulated systems as a function of time.

RMSD provides information about the overall stability of a protein complex with regard to variance from the initial structure. All selected systems were considerably stable, with an estimated RMSD of about 1.4 Å (Figure 6). Upon estimation of the average variance, **ZINC000095542577**, **ZINC00000049899562**, **ZINC00016933666** and **ZINC000028387333** demonstrated overall RMSDs of 1.472, 1.410, 1.379 and 1.359 Å, respectively, with a slight Cα backbone fluctuation of about 5000 ps that was stabilized in the remaining simulations. The study of the Cα backbone RMSD of **ZINC000095539404** yielded average RMSDs of 2.1432 Å. However, a tendency toward a slight drop was observed from 5000 to 8000 ps and was accompanied by significant variation, which indicated stability of the complexes. At 1000 and approximately 4000 ps, a marginal fluctuation was observed, which stabilized during the simulation. The data showed that all systems had stable internal motion.

### RMSF analysis

**Figure 7.**
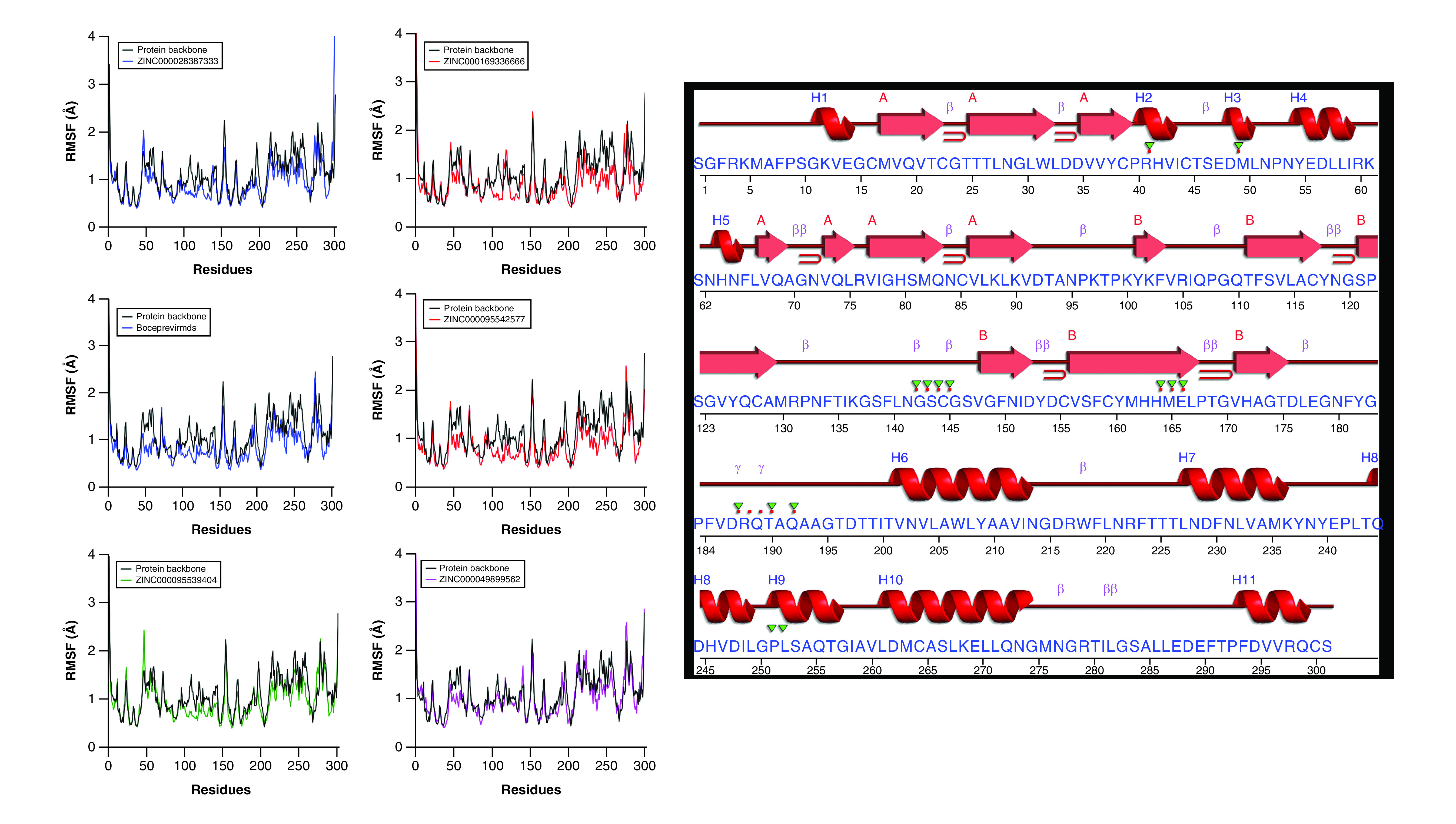
Calculation of the simulated systems of amino acid residues fluctuation (root mean square fluctuation).

RMSF of protein residues demonstrated a small amount of fluctuation on the backbone residues when binding the compound to the M^pro^ target. The RMSD values were determined by the least square fitting to the initial structure as a reference frame running over 100 ns trajectory. When no ligand was bound, the RMSF values of the M^pro^ protein residues were compared with the reference (Figure 7). Throughout all systems, the loop region fluctuated to a certain degree, whereas active residue regions remained unchanged during the simulation process. The results showed that the interaction of all five hits with the target protein led to their stabilization. Binding of the five chosen compounds in the active site domain caused a decrease in fluctuations with respect to protein structure fluctuations on the loop, whereas binding in the active site domain of all chosen compounds did not result in additional fluctuations in the protein structure. In addition, these fluctuations were observed primarily on the third to sixth and eighth helices. It can be assumed that no backbone conformational changes took place in the M^pro^ during ligand binding. Furthermore, the active binding domain was on the second, third, eighth and ninth helix regions. Notably, a sharp peak was seen most significantly on the close residue from 142 to 164.

### RoG & binding analysis of different ligands with M^pro^

**Figure 8.**
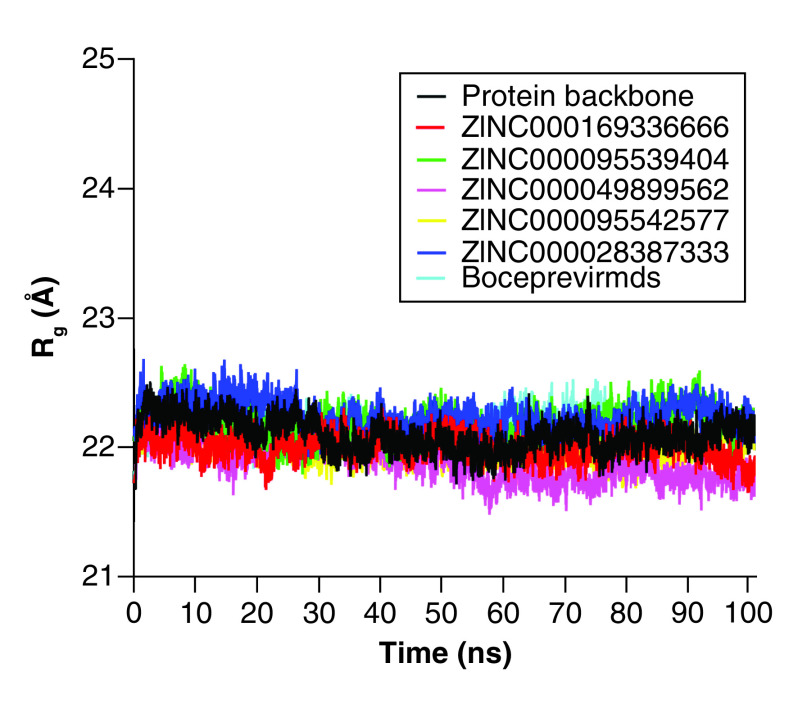
Measurement of radius of gyration of the simulated systems as a function of time.

RoG after ligand binding provides information about the folding of the protein structure. Consequently, RoG was evaluated to assess the system's compactness over time. Less compact protein (less folded) with high conformational entropy was seen with higher RoG values, whereas more folded protein with higher structural stability was associated with low RoG values. As shown in Figure 8, the average gyration scores of all systems were between 21.5 and 24 Å (Table 3). The simulation data revealed that all of the systems were compact, suggesting that the systems converged well.

**Table 3.** Average gyration scores for selected compounds.

Compound	Average gyration score
Protein backbone	22.099 ± 0.126
**ZINC000169336666**	22.006 ± 0.100
**ZINC000028387333**	22.249 ± 0.113
**ZINC000095539404**	22.187 ± 0.123
**ZINC000049899562**	21.890 ± 0.146
**ZINC000095542577**	21.984 ± 0.104
Boceprevir	22.154 ± 0.111

**Figure 9.**
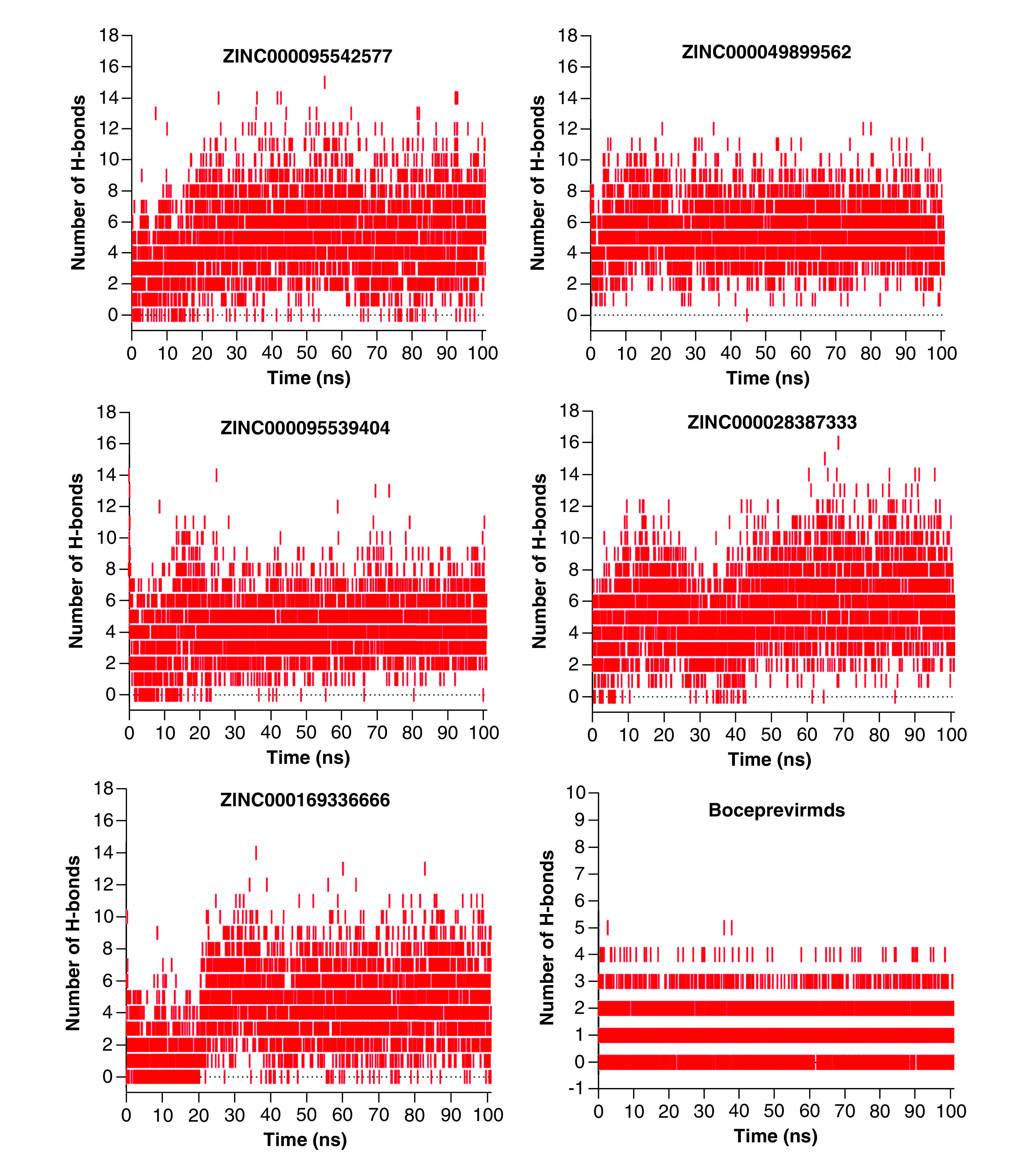
Stabilization of hydrogen bond over a 100-ns production run.

A simulation of hydrogen bond stability was conducted to further validate the stability of the hit compounds. This was performed by calculating all possible hydrogen bond donors and acceptors in the active site domain of the 3CLpro protein. The hydrogen bond formations between the donors and acceptors were subjected to a fixed geometric criterion of ≤3.5 Å and a fixed angle of 30° between the donors and acceptors. Figure 9 shows the hydrogen bond formation between the hit compounds and the active residues, which were completely preserved during the MD simulation run. As shown in Figure 8, a maximum of 16 hydrogen bonds were observed for **ZINC000028387333** with the 3CLpro protein. Compound **ZINC000095542577** also preserved 15 hydrogen bonds within the active site domain. In addition, compounds **ZINC000049899562**, **ZINC000095539404** and **ZINC000169336666** maintained 14 hydrogen bonds within the active site domain. In addition to the stability of the hydrogen bond formations, compounds **ZINC000169336666**, **ZINC000049899562**, **ZINC000095539404**, **ZINC000028387333** and **ZINC000095542577** showed smaller RMSDs of around 1.79, 1.73, 1.76, 1.92 and 1.92 Å, respectively (Table 4). The findings demonstrated that the binding of these five compounds to the active amino acids was located predominantly in the loop region, with some of the compounds present in the active pockets. The screening hits for this study resulted in a diversity of chemical compounds.

**Table 4.** Average root mean square deviation for selected compounds.

Compound	Average RMSD
Protein backbone	1.939 ± 0.225
**ZINC000169336666**	1.786 ± 0.442
**ZINC000028387333**	1.922 ± 0.300
**ZINC000095539404**	1.760 ± 0.290
**ZINC000049899562**	1.731 ± 0.225
**ZINC000095542577**	1.924 ± 0.249
Boceprevir	1.337 ± 0.243

RMSD: Root mean square deviation.

### ADMET study

**Figure 10.**
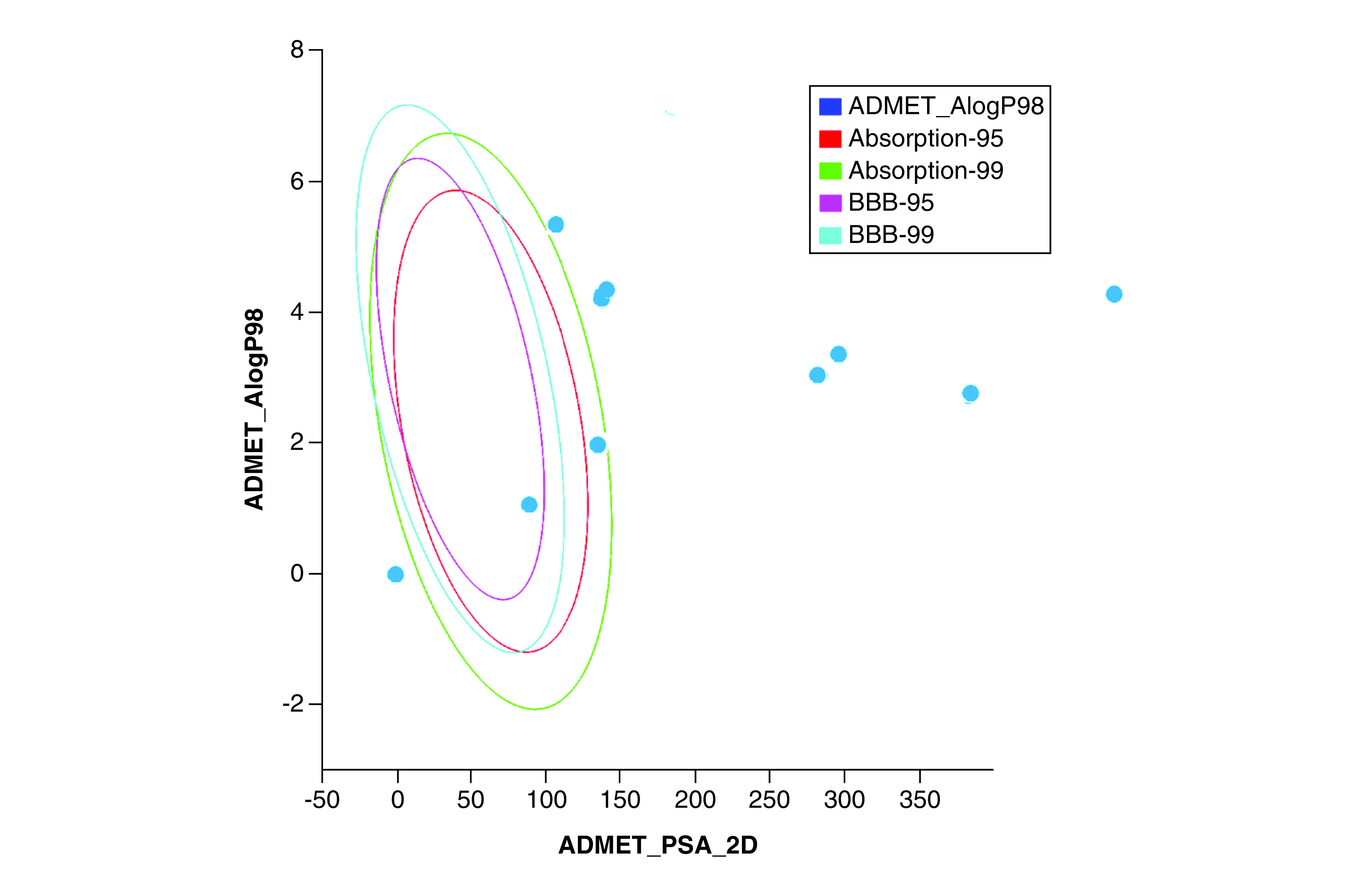
Polar surface area versus AlogP for the selected hits demonstrating the 95% and 99% confidence threshold ellipses aligned with intestinal absorption and blood–brain barrier.

Pharmacokinetics and toxicity effects play essential roles in choosing lead compounds and designing safe and effective compounds. Therefore, ADMET properties of the selected candidates were analyzed. To determine the compounds' drug-like properties, Lipinski's rule of five [43] and Veber rules [44] were applied (Tables 5 & 6). As depicted in Table 5, BIOVIA Discovery Studio 4.5 was used to determine the ADMET parameters. Furthermore, an ADMET plot was constructed using the calculated AlogP98 versus 2D PSA. The plot indicated the confidence level of the predictions for the HIA and BBB penetration models. As shown in Figure 10, the plot that corresponded to the HIA and BBB penetration models demonstrated two analogous 95% and 99% confidence ellipses. The PSA value of a given compound was inversely proportional to the HIA value of any compound and thus displayed an inverse relationship with cell wall permeability [32]. AlogP98 was shown to be lipophilic. Moreover, since the value is a ratio, it can be used to estimate a compound's hydrophilicity and hydrophobicity. As a result, the hydrogen-bonding characteristics determined by calculating PSA may play a crucial role along with the AlogP98 calculation [30]. The ellipse with 95% confidence indicated a chemical boundary that included compounds with high absorption (90%). By contrast, the ellipse with 99% confidence showed the region of chemical boundary with compounds that had excellent absorption through the cell membrane. The criteria of PSA <140 Å2 and AlogP98 <5 should be fulfilled by any compound with optimum cell permeability.

**Table 5.** Absorption, distribution, metabolism, excretion and toxicity values of selected compounds.

Compound	Absorption level	CYP2D6	Hepatotoxicity	PPB	Solubility level	Solubility	BBBP level	AlogP98	2D PSA
**ZINC000095542577**	3	0	True	0	0	-15.7	4	2.756	384.46
**ZINC000028387333**	2	0	False	0	3	-3.91	4	5.346	104.68
**ZINC000095536495**	3	0	True	1	3	-3.91	4	6.639	179.94
**ZINC000029040250**	0	0	False	0	4	-1.75	3	1.03	89.97
**ZINC000003583397**	1	0	True	1	2	-4.22	4	1.976	139.41
**ZINC000049899562**	3	0	True	0	0	-9.61	4	3.392	295.78
**ZINC000169336666**	3	0	True	0	0	-9.35	4	3.009	283.89
**ZINC000014945752**	2	0	True	1	2	-4.97	4	4.235	136.58
**ZINC000014907365**	2	0	False	1	3	-3.14	4	4.372	141.87
**ZINC000095539404**	3	0	True	0	0	-15.6	4	2.756	384.46

AlogP98 must be <5 for good BBB absorption. Drug absorption values: 0 = good, 1 = moderate, 2 = low, 3 = very low and 4 = undefined. Hepatotoxicity indicates drug toxicity by predicted classes: false = nontoxic and true = toxic. CYP2D6 determines the inhibitory effect by known classes: 0 = noninhibitor and 1 = inhibitor. PPB: true = binding and false = nonbinding. Solubility predicts molar solubility of drugs with different ranges: level 0 or log(Sw) <-8.0 = extremely low; level 1 or -8.0 <log(Sw) <-6.0 = no, very low, but possible; level 2 or -6.0 <log(Sw) <-4.1 = yes, low; level 3 or -4.1 <log(Sw) <-2.0 = good solubility; level 4 or -2.0 <log(Sw) 0.0 = optimal solubility; and level 5 or 0.0 <log(Sw) = no, too soluble.

ADMET: Absorption, distribution, metabolism, excretion and toxicity; BBB: Blood–brain barrier; BBBP: Blood–brain barrier permeability; CYP2D6: Cytochrome P2D6; PPB: Plasma protein binding; PSA: Polar surface area.

**Table 6.** Physicochemical properties of ZINC compounds based on Lipinski's rule of five.

Compound	logP	Molecular weight	Num_H_acceptor	Num_H_donor	Molecular fractional PSA	Num_ring	Num_rotatable bond
**ZINC000095542577**	2.756	788.573	22	13	0.538	5	13
**ZINC000028387333**	5.346	633.776	5	3	0.159	6	13
**ZINC000095536495**	6.639	766.922	8	6	0.218	4	21
**ZINC000029040250**	-1.675	322.399	5	3	0.245	1	9
**ZINC000003583397**	2.204	421.812	7	4	0.434	3	6
**ZINC000049899562**	3.392	722.602	17	10	0.448	6	6
**ZINC000169336666**	3.009	720.587	17	9	0.445	7	3
**ZINC000014945752**	4.235	620.737	7	4	0.22	7	8
**ZINC000014907365**	4.372	554.674	7	4	0.238	3	16
**ZINC000095539404**	2.756	788.573	22	13	0.538	5	13

Num_H_Acceptor: Number of hydrogen bond acceptors; Num_H_Donor: Number of hydrogen bond donors; Num_Hring: number of rings; Num_rotatable bond: Number of rotatable bonds; PSA: Polar surface area.

Compound **ZINC000029040250** was selected for the ADMET study and showed good HIA (absorption level 0). Compound **ZINC000003583397** showed moderate absorption, whereas compounds **ZINC000014945752**, **ZINC000014907365** and **ZINC000028387333** had low absorption. The remaining compounds exhibited very low absorption. When investigating aqueous solubility, **ZINC000095543045**, **ZINC000169336666**, **ZINC000049899562** and **ZINC000095542577** showed extremely low aqueous solubility. Compounds **ZINC000003583397** and **ZINC000014945752** had low aqueous solubility. Compounds **ZINC000028387333**, **ZINC000095536495**, **ZINC000049899562**, **ZINC000169336666** and **ZINC000095539404** showed good aqueous solubility. Compound **ZINC000029040250** showed optimal solubility. Compounds **ZINC000095542577**, **ZINC000095536495**, **ZINC000003583397**, **ZINC000049899562**, **ZINC000169336666**, **ZINC000014945752** and **ZINC000095539404** showed probable hepatotoxicity. Compounds **ZINC000028387333**, **ZINC000029040250** and **ZINC000014907365** showed no hepatotoxicity. Compound **ZINC000029040250** demonstrated low plasma protein binding (level 3), whereas the other compounds showed undefined plasma protein binding (level 4). All selected compounds, with the exception of compounds **ZINC000029040250** and **ZINC000003583397**, were found to be outside of the AlogP98 versus 2D PSA confidence ellipse. All selected compounds, with the exception of **ZINC000029040250** and **ZINC000003583397**, fell outside the ADMET model ellipse filter, suggesting low intestinal absorption and BBB penetration capability. Figure 9 presents a plot of PSA and AlogP for selected compounds.

## Conclusion

SARS-CoV-2 M^pro^ is an interesting target for drug discovery. Several diverse compounds have been screened to explore SARS-CoV-2 M^pro^ inhibition utilizing pharmacophores [45], natural and bioactive compounds [46,47] as well as peptidomimetic inhibitors [48]. Based on the crystal structure of SARS-CoV-2 NSP5 protease, the ZINC database, which contains potential antiviral compounds, was virtually screened for potential NSP5 inhibitors. After the initial selection of 353 compounds out of 13,840, ten compounds were chosen using a PLIF approach, and five compounds were finally identified. The interaction between SARS-CoV-2 NSP5 and the selected hits was evaluated using MD simulation. RMSD and RMSF analyses and RoG calculation have all been used to analyze known antiviral drugs [19]. However, in this study, we used the ZINC database to virtually screen anti-NSP5 compounds.

Overall, this type of screening is valuable in the search for novel medicines. SARS-CoV-2 NSP5 is conserved compared with many mutated amino acids, including M902I, T6891M, S221W (GenBank MT039890.1); S247R (MT007544.1); I476V, P2079L, T5538I, A930V (MT050493.1); L3606F (MT276597.1); P1921S (MT121215.1); H3076Y (MT066176.1); G818S, F4321L, F797C (MT093571.1); P5828L, Y5865C (MT163719.1); and F3071Y (MT198652.2). Among the vast majority of SARS-CoV-2 isolates, NSP5 showed no mutation; however, the Vietnamese isolate (02Shuman2020VNM; National Center for Biotechnology Information reference: MT192773.1) showed R60C mutation.

To recapitulate, it can be concluded that compounds **ZINC000049899562**, **ZINC000169336666** and **ZINC000095542577** may act as anti-coronavirus agents. Computational studies proved that these compounds have great medicinal and pharmacological actions. Further experimental studies are needed to confirm these findings.

## Future perspective

The current study demonstrates the utilization of computer modeling and simulation for the field of drug discovery and development. The current COVID-19 pandemic continues to challenge health and economy. In addition, coronaviruses are well known for their ability to mutate and resist treatments and vaccines. Our study has aided in the identification of several potential anti-COVID-19 hits that can be considered the first step in developing potent antiviral drugs.

**Summary points**

- SARS coronavirus 2 (SARS-CoV-2) main protease is an interesting target for drug discovery.
- Computer modeling and simulation aided the discovery of potential hits.
- The ZINC antiviral database contains different chemical scaffolds.
- Virtual screening of the ZINC antiviral database yielded several hits with excellent binding interaction with the SARS-CoV-2 main protease.
- Molecular docking analysis aided in the identification of the potential hits.
- Molecular dynamics simulations were utilized to further analyze the hits.
- Three compounds with high binding interaction with NSP5 have been discovered.
